# Salivary Biomarkers of Oxidative Stress in Children with Chronic Kidney Disease

**DOI:** 10.3390/jcm7080209

**Published:** 2018-08-10

**Authors:** Mateusz Maciejczyk, Julita Szulimowska, Anna Skutnik, Katarzyna Taranta-Janusz, Anna Wasilewska, Natalia Wiśniewska, Anna Zalewska

**Affiliations:** 1 Department of Physiology, Medical University of Bialystok, Bialystok, 2c Mickiewicza Street, 15-233 Bialystok, Poland; 2 Department of Pedodontics, Medical University of Bialystok, 24a M. Sklodowskiej-Curie Street, 15-274 Bialystok, Poland; szulimowska.julita@gmail.com; 3 Students’ Scientific Group “Stomatological Biochemistry”, Department of Conservative Dentistry, Medical University of Bialystok, 24a M. Sklodowskiej-Curie Street, 15-274 Bialystok, Poland; anna.skutnik@o2.pl; 4 Department of Pediatrics and Nephrology, Medical University of Bialystok, 24a M. Sklodowskiej-Curie Street, 15-274 Bialystok, Poland; katarzyna.taranta@wp.pl (K.T.-J.); annwasil@interia.pl (A.W.); natalia.smalec@gmail.com (N.W.); 5 Department of Conservative Dentistry, Medical University of Bialystok, 24a M. Sklodowskiej-Curie Street, 15-274 Bialystok, Poland; azalewska426@gmail.com

**Keywords:** chronic kidney disease, salivary biomarkers, oxidative stress, oxidative damage

## Abstract

There are still missing non-invasive biomarkers of chronic kidney disease (CKD) in children. Therefore, the aim of the study was to evaluate oxidative stress indicators in the non-stimulated (NWS) and stimulated saliva (SWS) of CKD children (*n* = 25) and healthy controls (*n* = 25). Salivary antioxidants (catalase (CAT), peroxidase (Px), superoxide dismutase (SOD), uric acid (UA), reduced glutathione (GSH), albumin), redox status (total antioxidant capacity (TAC), total oxidant status (TOS), oxidative stress index (OSI)), and oxidative damage products (advanced glycation end products (AGE), advanced oxidation protein products (AOPP), malondialdehyde (MDA)) were evaluated. We have demonstrated the significantly higher activity of SWS GPx and SOD, as well as elevated concentrations of UA and albumin in NWS and SWS of CKD children vs. the control group. TAC, TOS and OSI were significantly higher only in SWS, while oxidative damage products (AGE, AOPP and MDA) were significantly higher in both NWS and SWS of CKD children. ROC analysis showed a considerably high diagnostic value of AOPP in both NWS and SWS of CKD children compared to controls (AUC = 0.92; 0.98). CKD is responsible for disturbances in salivary antioxidant systems and oxidative damage to proteins and lipids. Salivary AOPP can be a potential biomarker of CKD in children.

## 1. Introduction

In addition to arterial hypertension, diabetes and obesity, chronic kidney disease (CKD) is one of the most common civilization diseases [1]. CKD is a multi-symptomatic syndrome that occurs as a result of permanent reduction in the number of active nephrons (or their permanent damage) caused by pathological processes occurring within the kidney parenchyma [2]. Chronic kidney disease is classified in five stages depending on the reduction in the glomerular filtration rate (GFR)—Stage 1: >90 mL/min; Stage 2: 60–89 mL/min; Stage 3: 30–59 mL/min; Stage 4: 15–29 mL/min; and Stage 5: <15 mL/min [2]. This problem is particularly important in children, as CKD does not reveal any clinical signs for quite a long time [3]. Therefore, the disease is often diagnosed only in severe stages requiring renal replacement therapy. It is thus not surprising that CKD is sometimes described as a challenge to 21st-century paediatrics. The most common causes of the chronic kidney disease in children are urological defects (ca. 30%), glomerulopathies (ca. 25–30%), congenital nephropathies (ca. 20%) and kidney dysplasia (ca. 10%) [3]. In view of the constant increase in the number of children with CKD (including dialysis patients) and high economic costs of its therapy, new screening tests are being intensively sought to enable early and non-invasive laboratory diagnosis of this disease in children [1,3].

Recently, a significant influence of oxidative stress in the pathogenesis of chronic kidney diseases has been increasingly emphasized [4,5,6]. The term “oxidative stress” is defined as redox imbalances leading to oxidative damage to cell components (such as proteins, lipids and nucleic acids), and thus also to cell metabolism disorders that may ultimately result in cell death by apoptosis [7]. Oxidative stress is caused both by increased production of reactive oxygen species (ROS) and an impairment of the antioxidant defence mechanisms [8]. It has been demonstrated that the activation of the renin–angiotensin–aldosterone system in CKD patients disrupts nitric oxide (NO) production and stimulates NADPH (NOX) oxidase activity, which triggers the formation of free radicals and may be responsible for progressive renal fibrosis [9]. Moreover, due to the observed changes in enzymatic and non-enzymatic antioxidant systems as well as the accumulation of protein and lipid oxidation products in kidney parenchyma of CKD patients it is proposed to use oxidative stress biomarkers in diagnosing patients with CKD [6,9]. A particularly favourable diagnostic material in children is saliva as it is collected in a non-invasive, painless manner and does not require the participation of qualified medical personnel [10,11]. The usefulness of salivary biomarkers of oxidative stress has been demonstrated in the context of numerous systemic diseases (insulin resistance [12], obesity [13], diabetes [14], dementia [15]); however, there are still no studies to evaluate salivary redox markers in children with chronic kidney disease. Therefore, the aim of our study was to evaluate redox homeostasis, enzymatic and non-enzymatic antioxidants, and products of oxidative modifications in non-stimulated and stimulated saliva of children with CKD compared to healthy controls. Despite the clinical aspect, our research may also enable better understanding of the pathomechanisms of chronic kidney disease in children.

## 2. Materials and Methods

### 2.1. Patients

The research was approved by the local Research Ethics Committee of the Medical University of Bialystok, Poland (permission number R-I-002/43/2018). All the examined persons and/or their legal guardians agreed in writing to participate in the study.

The study included 25 CKD patients (15 boys and 10 girls) aged 7–18 (median 12.9 years of age) treated in the Department of Paediatrics and Nephrology of the University of Bialystok Children’s Clinical Hospital of L. Zamenhof. The causes of CKD in the patients included: urological defects (28%), glomerulopathies (28%), congenital nephropathies (20%), kidney dysplasia (12%), and undetermined aetiology (12%). CKD was defined and staged according to the Kidney Disease Improving Global Outcomes (KDIGO) criteria based on different Eger distribution: Stage 1: >90 mL/min/1.73 m^2^; Stage 2: 60–89 mL/min/1.73 m^2^; Stage 3: 30–59 mL/min/1.73 m^2^; Stage 4: 15–29 mL/min/1.73 m^2^; and Stage 5: <15 mL/min/1.73 m^2^. The estimated glomerular filtration rate (eGFR) was calculated using the updated Schwartz formula—eGFR (mL/min/1.73 m^2^) = 0.413 × (height in cm/s Cr) [2].

Blood pressure (BP) was measured by means of either the manual auscultatory technique or an automated oscillometric device after the subject had rested for 5 min in a sitting position. The average values of the second and third measurements of systolic BP and diastolic BP were used for subsequent analyses and based on a diagnosis concerning hypertension. According to its definition, hypertension occurred when the average value of the systolic and/or diastolic BP measurements were ≥95th percentile for age, gender, and height [16].

The control group (*n* = 25; 15 boys and 10 girls; median 12.9 years of age) consisted of generally healthy children selected by age and sex, among patients attending control visits at the Special Dental Clinic of the Medical University of Bialystok.

The criterion excluding a patient from the study and the control group was the occurrence of chronic systemic, metabolic, autoimmune and infectious diseases as well as liver, lung, thyroid and gastrointestinal diseases. Moreover, the study did not include people with poor oral hygiene (API > 20) and periodontal inflammation (SBI > 0.5, GI > 0.5) identified in the course of a dental examination. In addition, patients taking antibiotics, non-steroidal anti-inflammatory drugs, glucocorticosteroids, vitamins and dietary supplements were also not included in the experiment.

Upon the diagnosis of CKD, all patients were on a renal diet that was low in sodium and/or phosphorous and/or protein depending on patients’ condition and CKD stage [17]. Detailed characteristics of the patients and the control group are presented in Table 1.

### 2.2. Saliva Collection

Non-stimulated saliva (NWS) and stimulated saliva (SWS) were collected using the spitting method. The saliva was gathered after an all-night rest, always between 8 a.m. and 10 a.m. For at least 2 h before saliva collection, participants of the study and control groups did not consume any meals or drinks other than pure water, and did not perform any hygienic procedures within the oral cavity. In addition, the study subjects did not take any medications for at least 6 hours before saliva collection.

The saliva was collected on the first day after admission to hospital, always in the same child-friendly room, so that the patients did not feel uncomfortable or nervous. After at least 5 min of the adaptation period and two rinses of the oral cavity with distilled water at room temperature, the saliva was collected in a sitting position with the head slightly inclined downwards, and minimized movements of the face and lips. The saliva accumulated at the bottom of the oral cavity was spat into a sterile Falcon tube placed in an ice container. The collection time for NWS was 10 min; however, the saliva collected during the first minute was disposed of [13]. After a 5-min break, the collection of SWS was initiated. The stimulation of saliva secretion was performed by sprinkling 10 µL of 2% citric acid on the tip of the tongue every 30 s. SWS was collected for 5 min to a maximum volume of 5 mL, in the same manner as NWS [13]. The saliva volume was measured with a calibrated pipette with accuracy of 100 µL, and the flow of NWS and SWS was calculated by dividing the saliva volume by the time required to produce it. Immediately upon collection, the saliva was centrifuged (20 min, 3000× *g*, +4 °C; MPW 351, MPW Med. Instruments, Warsaw, Poland). In order to protect the samples from oxidation during their processing and storage, butylated hydroxytoluene (BHT, Sigma-Aldrich, Saint Louis, MO, USA; 10 μL 0.5 M BHT/1 mL of saliva) was added to the obtained supernatants [15,18]. The saliva samples for pH determination were analysed immediately upon collection (SevenMulti pH meter, Mettler-Toledo, Columbus, OH, USA). The samples of saliva for biochemical assays were frozen at −80 °C and were stored in these conditions until the performance of analyses (but not longer than six months).

### 2.3. Dental Examination

The dental examinations were performed in artificial lighting using a mirror, an explorer, and a periodontal probe in accordance with the criteria of the World Health Organization [19]. The examinations were always performed by the same dentist (JS) following the collection of NWS and SWS. The dental examination included the measurement of DMFT (decay, missing, filled teeth), SBI (Sulcus Bleeding Index) according to Műhemann and Son, GI (Gingival Index) according to Löe and Silness [20], and API (Approximal Plaque Index) according to Lange [15]. The DMFT index is the sum of teeth with caries (D), teeth extracted due to caries (M), and teeth filled as a result of caries treatment (F). The SBI showed the intensity of bleeding from the gingival sulcus after probing. GI criteria included qualitative changes in the gingiva, and API revealed the percentage of tooth surface with plaque [15].

Inter-rater agreements between the examiner (JS) and another experienced dentist (AZ) were assessed in 15 patients. The reliability for DMFT was *r* = 1.00; for SBI: *r* = 0.96; for GI: *r* = 0.98, and for API: *r*= 0.98.

### 2.4. Blood Collection

After an overnight fast, 9 mL of venous blood samples were collected in S-Monovette^®^ K3 EDTA blood collection system (Sarstedt, Nümbrecht, Germany). To separate plasma and erythrocytes, the samples were centrifuged (1500× *g*; 4 °C, 10 min). Erythrocytes were washed three times in cold 0.9% NaCl (*v*:*v*) and haemolysed by the addition of cold 50 mM phosphate buffer (pH 7.4) 1:9 (*v*:*v*) [21]. In order to prevent sample oxidation, 0.5 M butylated hydroxytoluene (Sigma-Aldrich, Nümbrecht, Germany; 10 μL/mL blood) was added [21]. All samples were stored at −80 °C (but not longer than six months).

### 2.5. Redox Assays

The performed analysis included: antioxidant enzymes (catalase (CAT, EC 1.11.1.6), salivary peroxidase (Px, EC 1.11.1.7), and superoxide dismutase (SOD, EC 1.15.1.1)), non-enzymatic antioxidants (uric acid (UA), reduced glutathione (GSH), and albumin), total antioxidant/oxidant status (total antioxidant capacity (TAC), total oxidant status (TOS), and oxidative stress index (OSI)), as well as oxidative damage products (advanced glycation end products (AGE), advanced oxidation protein products (AOPP), and malondialdehyde (MDA)). All parameters were analysed in the saliva samples. Enzymatic antioxidants were also estimated in erythrocytes, while non-enzymatic antioxidants, total antioxidant/oxidant status and oxidative damage products also in the blood plasma.

Unless stated otherwise, all assays have been performed in duplicate samples. The absorbance/fluorescence was measured using Infinite M200 PRO Multimode Microplate Reader from Tecan. All results were standardised to mg of total protein. The total protein concentration was determined via the method with bicinchoninic acid and bovine serum albumin as a standard (Thermo Scientific PIERCE BCA Protein Assay (Rockford, IL, USA)).

### 2.6. Salivary Antioxidants 

CAT activity was determined colorimetrically in triplicate samples by measuring the decomposition rate of hydrogen peroxide (H_2_O_2_) [22]. The absorbance was measured at 240 nm. One unit of CAT activity was defined as the amount of enzyme that decomposes 1 mmol H_2_O_2_ per minute.

Px activity was determined colorimetrically according to Mansson-Rahemtulla [23] based on the reduction of 5,5′-dithiobis-(2-nitrobenzoic acid) to thionitrobenzene acid. A decrease in the absorbance of thionitrobenzene acid was measured at 412 nm (five times at 30-s intervals).

SOD activity was determined colorimetrically in triplicate samples by measuring the cytosolic activity of SOD by inhibiting the oxidation of epinephrine to adrenochrome [24]. It was assumed that one unit of SOD activity inhibits the oxidation of epinephrine by 50%.

UA level was measured colorimetrically using the commercial kit (QuantiChrom™ Uric Acid DIUA-250; BioAssay Systems, Harward, CA, USA), as instructed by the manufacturer. In this method, 2,4,6-tripyridyl-s-triazine forms a blue complex with Fe^3+^ and UA. The absorbance was measured at 630 nm.

GSH concentration was determined colorimetrically using Ellman’s method with 5,5′-dithiobis-2-nitrobenzoic acid [25]. The absorbance was measured at 412 nm.

Albumin concentration was measured colorimetrically using bromocresol green (BCG) assay with bovine serum album as a standard [26]. The absorbance was measured at 628 nm.

### 2.7. Total Antioxidant/Oxidant Status

TAC level was measured colorimetrically in triplicate using 2,2-azinobis-3-ethylbenzothiazoline-6-sulfonic acid (ABTS) radical cation [27]. The absorbance was measured at 660 nm and calibration curve was prepared for 6-hydroxy-2,5,7,8-tetramethylchroman-2-carboxylic acid (Trolox).

TOS level was determined bichromatically (560/800 nm) based on the oxidation of Fe^2+^ to Fe^3+^ in the presence of the oxidants contained in the sample [28]. The results are expressed as micromolar H_2_O_2_ equivalent per litre.

Oxidative stress index (OSI) was calculated using the formula: OSI = TOS/TAC × 100 [12]. 

### 2.8. Oxidative Damage Markers

The AGE content was determined fluorimetrically by measuring the AGE-specific fluorescence at 350/440 nm [29]. Saliva samples were diluted in phosphate-buffered saline (pH 7.2) 1:5 (*v*:*v*).

The AOPP concentration was determined colorimetrically by measuring the oxidative capacity of the iodine ion at 340 nm [29]. Saliva samples were diluted in phosphate-buffered saline (pH 7.2) 1:5 (*v*:*v*) and a calibration curve was prepared for chloramine solutions.

The MDA concentration was measured colorimetrically using thiobarbituric acid reactive substances (TBARS) assay [30]. 1,1′,3,3′-tetraethoxypropane was used as a standard. The absorbance of the supernatants was measured at 535 nm.

### 2.9. Statistical Analysis

Statistical analysis was performed using the GraphPad Prism (GraphPad Software, La Jolla, CA, USA) and Statistica 10.0 system (StatSoft, Krakow, Poland). The Kolmogorov–Smirnov test showed no normal distribution of the obtained results, therefore nonparametric methods were implemented. The Mann–Whitney U test was used to analyze quantitative values between the study and control groups. The data was expressed as median, minimum, and maximum values. The associations between the measured parameters were tested by Spearman’s rank correlation coefficient. Statistical significance was established at *P* ≤ 0.05. Due to the lack of significant differences between the different types of CKD, the results of the redox assays were presented as CKD (all subgroups together) and a control group. The diagnostic value of the redox salivary biomarkers and the optimum cut-off values were determined based on receiver operating characteristic (ROC) analysis, known as the area under the curve (AUC).

## 3. Results

### 3.1. Patients

In patients with CKD we demonstrated considerably higher levels of serum creatinine, UA, urea, and 24-h urinary protein, albumin excretion compared to the controls. The detailed clinical characteristics of the patients are presented in Table 1.

We found no significant differences between oral hygiene indexes (APIs) and periodontal disease indexes (SBI, GI) in patients from both the study and control group (Table 2).

### 3.2. Salivary Gland Function

The secretion activity of salivary glands was analysed based on the measurement of salivary flow rate and the evaluation of total protein concentration and salivary amylase activity. We observed a significantly lower flow of non-stimulated (*P* = 0.0005) and stimulated (*P* = 0.0005) saliva in CKD patients compared to the controls. The content of total protein was considerably lower only in the non-stimulated saliva of patients from the study group vs. healthy children (*P* = 0.1442). The activity of salivary amylase was significantly lower in both non-stimulated (*P* < 0.0001) and stimulated saliva (*P* = 0.8901) of CKD patients in comparison with the control group (Table 3).

### 3.3. Salivary Antioxidants

**Figure 1:**
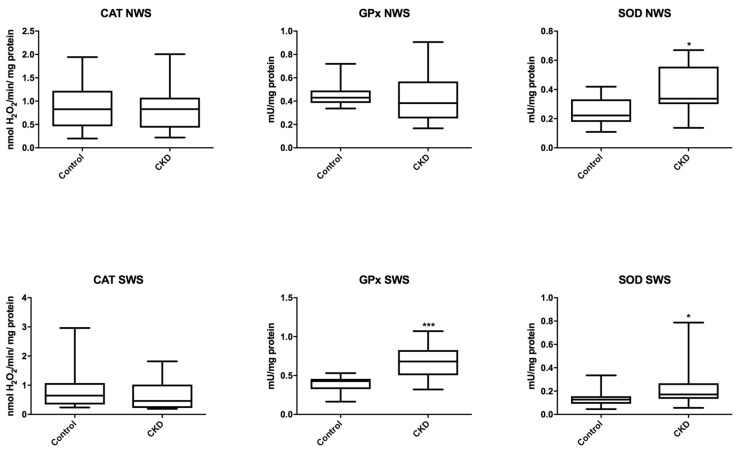
Salivary antioxidant enzymes in children with chronic kidney disease (CKD) and the controls. CAT, catalase; NWS, non-stimulated whole saliva; Px, salivary peroxidase; SOD, superoxide dismutase; SWS, stimulated whole saliva. Differences statistically important at: * *P* < 0.05, *** *P* < 0.0005.

In non-stimulated saliva of patients with CKD, the activity of antioxidant enzymes (CAT, Px, GR) did not differ significantly from the data obtained from the healthy controls. Only the SOD activity was noticeably higher in the study group (*P* = 0.0173). In stimulated saliva, the activity of Px and SOD was significantly higher in CKD patients (*P* = 0.0007; *P* = 0.0263 respectively), while the activity of CAT did not differ considerably from that of the control group (Figure 1).

**Figure 2:**
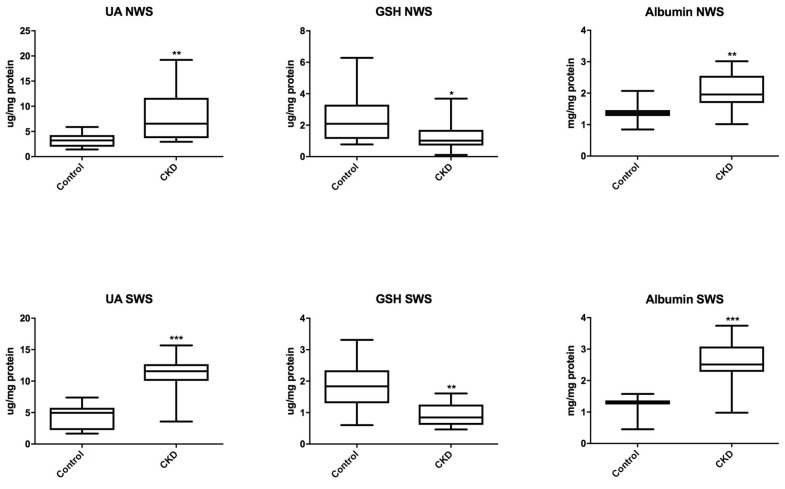
Salivary non-enzymatic antioxidants in children with chronic kidney disease (CKD) and the controls. GSH, reduced glutathione; NWS, non-stimulated whole saliva; SWS, stimulated whole saliva. Differences statistically important at: * *P* < 0.05, ** *P* < 0.005, *** *P* < 0.0005.

We demonstrated a significant increase in the concentration of UA and albumin both in non-stimulated (*P* = 0.0043; *P* = 0.0100) and stimulated saliva (*P* < 0.0001; *P* = 0.0002) in patients with CKD than in the controls. However, CKD patients showed significantly lower concentration of GSH both in non-stimulated (*P* = 0.0299) and stimulated saliva (*P* = 0.0027) compared to the healthy controls (Figure 2).

### 3.4. Total Antioxidant/Oxidant Status

**Figure 3:**
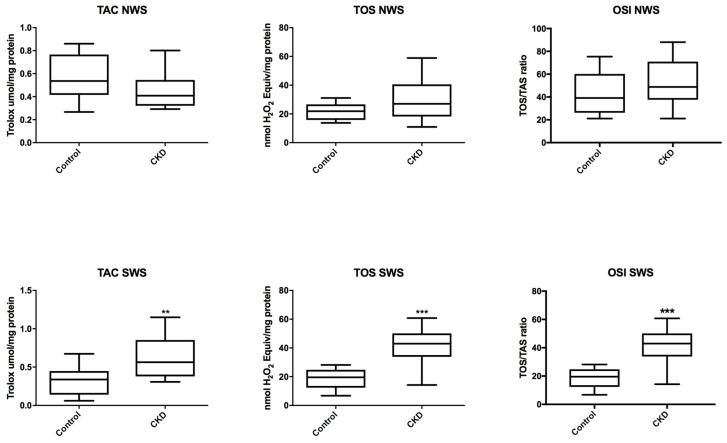
Salivary total antioxidant/oxidant status in children with chronic kidney disease (CKD) and the controls. NWS, non-stimulated whole saliva; OSI, oxidative stress index; SWS, stimulated whole saliva; TAC, total antioxidant capacity; TOS, total oxidative status. Differences statistically important at: ** *P* < 0.005, *** *P* < 0.0005.

In non-stimulated saliva of CKD patients, TAC, TOS and OSI were at similar levels as in the control group, while in stimulated saliva we observed a significant increase in TAC, TOS and OSI in patients with CKD vs. healthy children (*P* = 0.0054; *P* < 0.0001; *P* < 0.0001) (Figure 3).

### 3.5. Oxidative Modification Products

**Figure 4:**
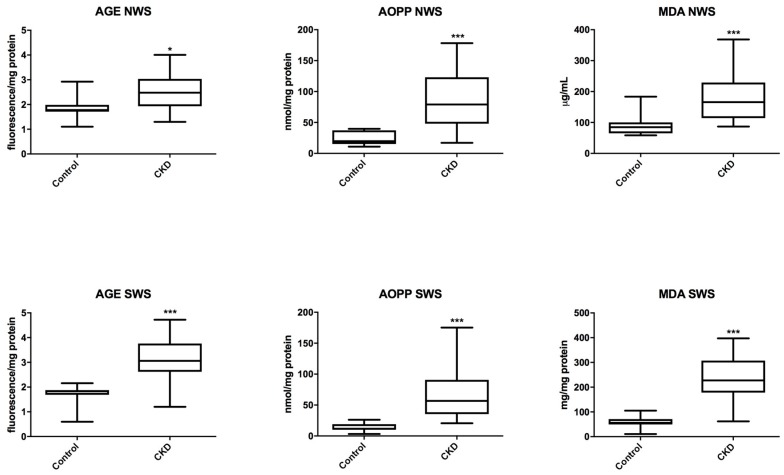
Salivary markers of oxidative damage in children with chronic kidney disease (CKD) and the controls. AGE, advanced glycation end products; AOPP, advanced oxidation protein products; MDA, malondialdehyde; NWS, non-stimulated whole saliva; SWS, stimulated whole saliva. Differences statistically important at: * *P* < 0.05, *** *P* < 0.0005.

In patients with CKD we observed a significant increase in the concentration of the markers of oxidative protein (AGE and AOPP) and lipid (MDA) damage both in non-stimulated saliva (*P* = 0.0235; *P* = 0.0003; *P* = 0.0007) and in stimulated saliva (*P* = 0.0002; *P* < 0.0001; *P* < 0.0001) compared to the controls (Figure 4).

### 3.6. Erythrocytes and Plasma Redox Markers

Similarly to saliva, we have observed a statistically higher level of plasma UA and albumin as well as lower plasma GSH in CKD children vs. control group. The activity of enzymatic antioxidants did not differ statistically in the erythrocytes of CKD patients with the exception of SOD activity. TAC and OSI were significantly elevated in patients in the study group. We also demonstrated a significantly higher concentration of oxidative damage markers (AGE, AOPP and MDA) in children with CKD compared to controls (Table 4).

### 3.7. Correlations

**Figure 5:**
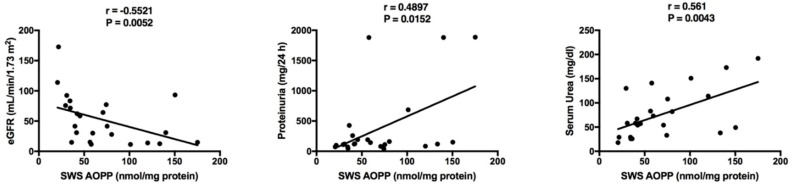
Correlations between salivary advanced oxidation protein products (AOPP) and clinical parameters in children with chronic kidney disease (CKD). eGFR, estimated glomerular filtration rate using Schwartz formula; SWS, stimulated whole saliva.

The results of statistically significant correlations are presented in Table 5 and Table 6. Interestingly, a negative correlation between SWS AOPP and eGFR was found in children with chronic kidney disease (Table 5). In this group we also demonstrated a positive correlation between SWS AOPP and proteinuria, as well as SWS AOPP and serum urea (Figure 5). Additionally, most salivary redox biomarkers (UA, albumin, TAC, TOS, AOPP and MDA) correlated with their level in the blood plasma (Table 6).

### 3.8. ROC Analysis

The results of ROC analysis for the evaluated oxidative stress parameters are presented in Table 5. Interestingly, ROC analysis showed a considerably high diagnostic value of AOPP assay in both non-stimulated and stimulated saliva in children with CKD compared to the healthy controls. The optimum AOPP concentration (AUC 0.92, *P* = 0.0003) in NWS differentiating the two groups was the value above 37.85 nmol/mg of protein, at which sensitivity was 81.25%, and specificity 81.82%. The optimum SWS AOPP concentration (AUC 0.98, *P* < 0.0001), differentiating CKD patients from the control group, was >25.58 nmol/mg of protein, at which sensitivity was 92.00%, and specificity 92.31% (Table 7).

## 4. Discussion

Our study is the first to evaluate the diagnostic utility of salivary biomarkers of oxidative stress in children with chronic kidney disease. We have demonstrated that CKD causes disorders within enzymatic and non-enzymatic antioxidant systems as well as increased oxidative damage of salivary proteins and lipids. We point out that salivary parameters of redox homeostasis (particularly AOPP) may be potential diagnostic biomarkers of chronic kidney disease in children.

With the development of sensitive analytical methods we can observe an increased interest in the use of other body fluids than blood in the diagnosis of chronic systemic diseases. Non-invasive collection of test material plays an important role in reducing patients’ anxiety associated with this procedure (especially in children and the disabled) and may contribute to more frequent performance of control tests. In the therapeutic process, early diagnosis is crucial in enabling quick recognition of the disease and applying appropriate therapy. An interesting alternative to blood, commonly used in diagnosis, is saliva—the exudation of large salivary glands (parotid, submandibular and sublingual) as well as numerous smaller glands located in the oral mucosa [15]. Saliva consists mainly of water (99%), and also proteins, electrolytes and α-amylase; however, its final composition depends on the type of salivary glands in which it is produced [31]. Non-stimulated whole saliva (NWS) is produced mostly by the submandibular (60%) and parotid glands (20%), but upon stimulation the share of submandibular saliva decreases (to about 50%) in favour of the parotid gland saliva (40%) [32,33]. In general, chemical substances included in saliva can be divided into two groups: compounds produced exclusively in salivary glands and those transported from plasma to saliva. The latter group is particularly important for laboratory diagnosis, as numerous components of saliva reflect their concentration in serum [11]. The advantages of the use of saliva in laboratory diagnosis also include the low cost, ease, and non-invasive nature of sample collection as well as relatively long shelf life of saliva compared to blood [34]. Thus, saliva is used for the diagnosis of cancer as well as cardiovascular, autoimmune, infectious and metabolic diseases, and in the monitoring of drugs and/or intoxicants concentration [11,34].

Chronic kidney disease is a major clinical problem not only in the adult population but also, above all, in children and adolescents [1]. Due to its asymptomatic nature, the disease is diagnosed with a long delay, which hinders its medical prognosis and advances the necessity of starting renal replacement therapy [3]. Unfortunately, there are no non-invasive CKD markers to serve as screening tests in children. The most commonly used indicators of kidney damage like: serum creatinine concentration, eGFR, albuminuria, proteinuria, and imaging techniques, such as medical ultrasound or CT scans, are not optimal to detect kidney function impairment in early stages [3]. Since oxidative stress plays a significant role in the pathogenesis of CKD, redox homeostasis biomarkers are proposed in the diagnosis of chronic kidney disease [5]. In adults with CKD, decreased concentrations of glutathione, one of the most important intracellular antioxidants, were observed along with increased concentration of UA and plasma markers of oxidative damage to proteins and lipids (↑AOPP, ↑MDA) [35]. However, there are no available data on the salivary parameters of oxidative stress in children with CKD. Our study has been the first to evaluate the activity of salivary antioxidant enzymes, concentrations of non-enzymatic antioxidants and free radical scavengers, total antioxidant/oxidant capacity, and concentration of oxidative modification products. Generally, in children with CKD we demonstrated the intensification of salivary mechanisms of antioxidant protection (↑Px, ↑SOD, ↑GR, ↑UA, ↑albumin), which suggests an adaptive reaction of the body to increased ROS production (↑TOS, ↑OSI). It should be noted that the total antioxidant activity is not a simple component of individual antioxidants. Total antioxidant/oxidant status assessment provides more information as it includes the interactions between antioxidants, and is the resultant of the biological system activity aimed at neutralisation of free radicals [36]. Therefore, despite a significant decrease in GSH concentration, the redox balance in saliva is rather shifted in favour of antioxidant processes (↑TAC, ↑FRAP). However, oxidative damage to proteins (AGE, AOPP) and lipids (MDA) occurs under these conditions, which suggests that the body cannot fully prevent oxidative damage (↑OSI). Antioxidant supplementation may be advisable in children with CKD, but this matter requires further research.

UA plays an important role in the overall antioxidant potential. It is believed that this compound determines 70–85% of antioxidant capacity of plasma or saliva [27]. From the physiological perspective, UA is formed by catabolism of purine bases, and its formation involves xanthine oxidase (XO)—enzyme belonging to the class of oxidoreductases [37]. In addition to NADPH oxidase (NOX), XO is one of the primary sources of free radicals in the cell [7]. The increased formation of UA can therefore generate significant amounts of ROS. Uric acid itself, in addition to its antioxidant properties, can also demonstrate pro-oxidant effect [38]. The UA has been proven to generate free radicals, inter alia in the reaction with peroxynitrite [37]. Moreover, it has been demonstrated that in the course of hyperuricemia the inflammation (↑inflammation markers) or extracellular matrix remodelling may intensify, which results in increased UA concentration in connection with the pathogenesis of hypertension, insulin resistance, metabolic syndrome, cardiovascular diseases, or stroke [39,40,41]. In the kidneys, however, UA may activate the renin-angiotensin-aldosterone system, which is an important source of ROS responsible for endothelial dysfunction and mesenchymal-epithelial transformation [41,42]. Thus UA may be responsible for the development of atherosclerotic lesions in the afferent artery as well as interstitial renal hypertension that form the pathological background of CKD [43]. In our study, the increase in concentration of salivary UA correlated with kidney damage markers (GFR and proteinuria), which may indicate the contribution of uric acid to the progression of CKD. However, it should be remembered that UA is a non-specific antioxidant; its higher level may also reflect the hyperuricemia.

A significant part of the study was to evaluate the diagnostic usefulness of salivary biomarkers of oxidative stress in diagnosing CKD in children. ROC analysis has shown that salivary advanced oxidation protein products (AOPP) may constitute a particularly interesting parameter. At high sensitivity and specificity we observed that AOPP very accurately differentiate between children with CKD and healthy controls (AUC in NWS = 0.92, and in SWS = 0.98). Correlations between AOPP and eGFR, urine protein and serum urea also prove the usefulness of salivary AOPP. Although AOPP are used as indicators of various disease units (diabetes [14], insulin resistance [31], cardiovascular diseases [44], dementias [15]), they were first described as markers of inflammation (activation of macrophages and neutrophils) and kidney damage in patients with uremia [35]. It is believed that increased oxidative stress leads to the formation of AOPP that may accumulate in the kidneys, leading to end-stage renal disease (ESRD) [45,46]. The accumulation of AOPP has been observed to increase the expression of renin-angiotensin-aldosterone system proteins, impairs the activity of endothelium (NO secretion) and activates NOX, which further increases ROS production [45,47]. Advanced glycation end products (AGE) reveal a similar effect [48]. AOPP and AGE may induce the synthesis of proinflammatory cytokines through the NF-κB dependant pathway as well as play an important role in the progression of proteinuria and hardening of renal glomeruli, thus reducing the number of podocytes and triggering their apoptosis [47,49]. Although AOPP are described as markers of kidney damage [45,46], our study has been the first to indicate the usefulness of salivary AOPP for the diagnosis of CKD. We believe that the lack of differences between the redox markers evaluated in children at different CKD stages as well as in dialyzed vs. non-dialyzed children may result from a relatively small number of patients qualified for the study. It is well known that the dialysis procedure can affect serum creatinine and GFR levels as well as enhance oxidative stress level independent of the renal failure. Therefore, it is advisable to perform further examinations with a larger group of patients (dialyzed and non-dialyzed), especially as our results clearly indicate the usefulness of salivary redox biomarkers in diagnosing chronic kidney disease.

The use of saliva as a diagnostic material also involves certain limitations. In some cases, local pathological processes (such as caries, periodontitis) may affect the evaluated oxidative stress parameters. Therefore, patients whose dental examinations revealed poor oral hygiene (API > 20) and gingivitis (SBI < 0.5, GI < 0.5) were excluded from our experiment. Furthermore, the assessed oxidative stress markers are not exclusively specific to kidney inflammatory diseases. However, on the other hand, our study indicates the adequacy of the evaluation of redox parameters in saliva, which may be an alternative diagnostic material for the blood. We showed that the most salivary biomarkers correlated with their level in the blood plasma, and therefore, saliva may be considered in laboratory diagnostics of CKD in children.

Disturbances of redox homeostasis observed in the study may result not only from the intensity of the disease process, but also from hypofunction of salivary glands in the course of CKD. Importantly, we have demonstrated reduced non-stimulated and stimulated saliva secretion in children with CKD as well as significantly lower activity of salivary amylase, which is considered the most important marker of salivary gland secretion activity [32]. A decrease in saliva production in the oral cavity may be responsible for an increased incidence of caries, periodontal diseases or fungal infections, as these conditions are far more common in CKD patients [50]. The proteins found in saliva (acidic and alkaline glycoproteins, statherins, histatins, lactoferrin and lysozyme) inhibit the formation of antibacterial platelets, balance the processes of demineralization and remineralization of tooth enamel, and serve antibacterial, antifungal and buffering functions [32]. The increased activity of antioxidant enzymes in the stimulated saliva compared to NWS is not surprising due to the fact that the parotid gland (the primary source of SWS) is the main place of salivary antioxidants formation [33]. However, we did not observe any correlation between salivary flow rate, salivary amylase activity and redox parameters, which may indicate the lack of relationship between the secretion activity of salivary glands and increased oxidative stress intensity.

Finally, it is also worth noting certain limitations connected with the study. We evaluated only selected (though most commonly used) oxidative stress parameters, therefore we could not fully characterize the redox equilibrium in patients with CKD. Moreover, most pathological processes in the course of CKD occur in kidney, which reduces the evaluation of salivary redox biomarkers to an auxiliary value. In addition, the observed changes in endogenous antioxidant systems may be disturbed by the implemented pharmacological treatment (pro-oxidant iron or antioxidant ACE inhibitors), increased ferritin level as well as may also result from hypofunctions of salivary glands during CKD. However, it has been the first study to evaluate salivary redox biomarkers in children with CKD and it has indicated their potential use in laboratory diagnosis. The study was attended by carefully selected patients with a healthy oral cavity, without most other systemic diseases.

## 5. Conclusions

CKD is associated with disorders within salivary antioxidant systems, and oxidative damage to proteins and lipids. The salivary parameters of oxidative stress, especially AOPP, may serve as potential biomarkers of CKD as alterative to the blood. CKD leads to impaired secretion activity of salivary glands in children, resulting in a decrease in salivary flow rate, total protein, and α-amylase. Antioxidant supplementation should be considered in children with CKD.

## Figures and Tables

**Table 1:** Clinical and laboratory data of children with chronic kidney disease (CKD) and healthy controls.

Patient Characteristic		Stage 1	Stage 2	Stage 3	Stage 4	Stage 5	All CKD Patients	Control Group
Sex	Male *n*	1	5	5	2	4	17	17
	Female *n*	2	3	2	1	1	9	9
Age		16.75 (9.25–17.75)	14.03 (9.25–18.00)	11.50 (7.25–15.50)	13.00 (9.25–14.50)	10.50 (6.50–17.80)	12.90 (6.50–18.00)	12.90 (6.50–18.00)
Weight (kg)		53.00 (36.80–59.00)	51.25 (23.90–75.00)	34.00 (25.50–49.50)	22.70 (22.00–39.00)	26.00 (19.30–60.00)	34.30 (19.30–75.00)	37.30 (19.00–71.00)
Height (cm)		161.50 (135.50–166.00)	154.50 (126.00–168.00)	131.50 (114.50–163.00)	129.00 (125.00–150.00)	130.00 (102.00–164.00)	136.80 (102.00–168.00)	135.20 (100.00–173.00)
Systolic BP (mmHg)		112.00 (97.00–136.00)	116.50 (101.00–140.00)	117.00 (96.00–125.00)	112.00 (109.00–116.00)	111.00 (108.00–139.00)	114.00 (96.00–140.00)	106.00 (100.00–120.00)
Diastolic BP (mmHg)		68.00 (63.00–80.00)	67.00 (56.00–80.00)	73.00 (60.00–77.00)	75.00 (74.00–77.00)	80.00 (64.00–94.00)	73.50 (56.00–94.00)	69.00 (55.00–77.00)
Hypertension *n*		1	1	2	1	3	8	0
Dialysis *n*		0	0	0	1	5	6	0
Albuminuria (mg/24 h)		9.15 (8.71–10.62)	11.18 (8.40–135.10)	238.50 (150.20–310.90)	345.50 (238.50–626.40)	1002.00 (750.20–1576.00)	150.20 (8.40–1576.00) *	7.15 (5.15–9.23)
Proteinuria (mg/24 h)		91.50 (84.00–99.00)	191.50 (99.00–1109.00)	123.70 (48.30–1886.00)	1172.00 (686.00–1411.00)	1428.00 (1172.00–1882.00)	192.50 (48.30–1886.00) *	55.50 (25.15–89.00)
eGFR (mL/min/1.73 m^2^)		113.90 (93.20–172.70)	76.50 (64.18–89.40)	41.60 (27.90–52.20)	15.30 (15.50–17.90)	11.60 (10.50–13.90)	51.39 (10.50–172.70) *	120.00 (110.00–180.00)
Serum Cr (mg/dL)		0.95 (0.43–1.02)	0.97 (0.75–1.66)	1.66 (1.22–2.66)	4.03 (2.66–4.11)	7.23 (6.16–8.93)	1.59 (1.43–8.93) *	0.55 (0.30–0.65)
Serum Urea (mg/dL)		29.00 (18.00–54.00)	51.50 (26.00–130.00)	58.00 (26.00–141.00)	114.00 (93.00–141.00)	157.00 (108.00–192.00)	62.50 (18.00–192.00) *	15.00 (13.50–36.00)
Hemoglobin (g/dL)		13.00 (11.70–16.10)	13.55 (10.60–16.70)	11.50 (10.60–15.70)	10.50 (10.40–10.90)	11.70 (9.20–13.00)	11.70 (9.20–16.70)	14.10 (13.20–15.00)
Hematocrit (%)		38.00 (36.60–47.50)	38.00 (31.50–48.70)	36.20 (29.70–40.80)	31.00 (35.50–31.70)	35.30 (26.00–38.70)	36.35 (26.00–48.70)	39.00 (35.5–45.00)
Iron (µg/dL)		28.50 (22.00–35.00)	84.00 (22.00–132.00)	57.00 (40.00–105.00)	82.00 (80.00–90.00)	89.00 (67.00–161.00)	82.00 (22.00–161.00)	79.00 (43.00–154.00)
Ferritin (ng/mL)		32.55 (25.00–40.10)	40.10 (26.32–264.80)	63.12 (11.63–291.60)	291.60 (12.84–332.60)	320.20 (33.12–386.70)	53.26 (11.63–386.70) *	25.5 (17.00–34.00)
ALP (U/L)		67.00 (64.00–197.00)	201.00 (64.00–346.00)	188.50 (124.00–284.00)	160.00 (72.00–487.00)	196.00 (106.00–531.00)	188.50 (64.00–531.00) *	45.5 (20.00–85.00)
Ca^2+^ (mmol/L)		2.47 (2.41–2.61)	2.40 (1.95–2.61)	2.45 (2.18–264)	2.35 (2.34–2.40)	2.48 (2.30–2.67)	2.41 (1.95–2.67)	2.30 (2.20–2.55)
PTH (pg/mL)		36.10 (33.40–64.20)	47.00 (24.30–117.00)	50.90 (34.10–123.80)	34.70 (24.10–125.50)	84.00 (27.00–121.00)	52.90 (24.10–125.50) *	36.00 (8.52–51.50)
Drugs	iron *n*	1	1	2	1	2	7	0
	loop diuretics *n*	0	2	2	2	3	9	0
	ACEI *n*	1	1	2	1	2	7	0
	β-blockers *n*	0	1	1	1	2	5	0
	CCB *n*	0	0	0	0	1	1	0

ACEI, angiotensin-converting-enzyme inhibitors; ALP, alkaline phosphatase; BP, blood pressure; Ca^2+^, calcium ions; CCB, calcium channel blockers; Cr, creatinine; eGFR, estimated glomerular filtration rate using Schwartz formula; PTH, parathyroid hormone; UA, uric acid. Significant changes vs. control: * *P* < 0.05.

**Table 2:** Dental examination of children with chronic kidney disease (CKD) and healthy controls.

Characteristic	Control *n* = 25	CKD *n* = 25
DMFT	4.00 (0.00–6.00)	4.00 (0.00–8.00)
SBI	0.00 (0.00–0.05)	0.00 (0.00–0.05)
GI	0.00 (0.00–0.05)	0.00 (0.00–0.05)
API	5.00 (10.00–18.00)	6.00 (12.00–19.00)

API, Approximal Plaque Index; DMFT, decay, missing, filled teeth; GI, Gingival Index; SBI, Sulcus Bleeding Index.

**Table 3:** Non-stimulated and stimulated salivary flow, total protein, amylase and pH.

Parameter	NWS		SWS	
	Control *n* = 25	Study Group *n* = 25	Control *n* = 25	Study Group *n* = 25
Salivary flow (μL/min)	0.43 (0.33–0.33)	0.25 (0.10–0.50) *	1.40 (1.00–1.90)	0.80 (0.20–1.60) *
Total protein (μg/mL)	1759.00 (1103.00–2753.00)	1248.00 (797.70–2768.00)	1741.00 (1482.00–3193.00)	1001.00 (654.70–2477.00) *
Salivary amylase (μmol/mg protein)	0.23 (0.16–0.41)	0.07 (0.02–0.17) *	0.25 (0.12–0.81)	0.20 (0.07–0.47) *
Salivary pH	7.56 (7.12–7.94)	7.77 (7.32–8.21)	7.22 (6.26–7.90)	7.40 (5.35–8.18)

NWS, non-stimulated whole saliva; SWS, stimulated whole saliva. Significant changes vs. control: * *P* < 0.05.

**Table 4:** Erythrocytes and plasma antioxidants, total antioxidant/oxidant status and oxidative damage in children with chronic kidney disease (CKD) and the controls.

Parameter	Control	CKD	*P*
*Non-enzymatic antioxidants*			
UA (μg/mg protein)	0.52 (0.27–1.39)	5.05 (3.80–6.92)	<0.0001
GSH (μg/mg protein)	3.67 (2.01–5.77)	1.23 (0.37–4.12)	0.0056
Albumin (mg/mg protein)	2.19 (1.03–3.26)	2.71 (2.06–4.50)	0.0241
*Enzymatic antioxidants*			
CAT (nmol H_2_O_2_/min/mg protein)	0.28 (0.04–0.66)	0.24 (0.05–0.87)	0.8032
Px (mU/mg protein)	0.28 (0.22–0.44)	0.32 (0.26–0.65)	0.1101
SOD (mU/mg protein)	0.05 (0.02–0.13)	0.09 (0.02–0.12)	0.0235
*Total antioxidant/oxidant status*			
TAC (Trolox μmol/mg protein)	0.11 (0.01–0.44)	0.35 (0.03–1.52)	0.0012
TOS (nmol H_2_O_2_Equiv/mg protein)	9.99 (4.54–21.82)	13.98 (6.76–25.04)	0.2703
OSI (TOS/TAC ratio)	33.97 (13.15–405.8)	81.67 (22.16–389.2)	0.0122
*Oxidative damage products*			
AGE (fluorescence/mg protein)	0.74 (0.36–1.13)	1.23 (0.73–1.90)	<0.0001
AOPP (nmol/mg protein)	5.26 (2.68–12.17)	10.50 (6.49–19.83)	<0.0001
MDA (μmol/mg protein)	207.20 (109.30–327.00)	267.40 (151.00–611.20)	0.0387

AGE, advanced glycation end products; AOPP, advanced oxidation protein products; CAT, catalase; GSH, reduced glutathione; MDA, malondialdehyde; Px, salivary peroxidase; SOD, superoxide dismutase; TAC, total antioxidant capacity; TOS, total oxidant status; UA, uric acid.

**Table 5:** Correlations between oxidative stress biomarkers and clinical parameters in children with chronic kidney disease (CKD).

Pair of Variable	*r*	*P*
NWS GSH & Albuminuria	0.872	0.011
NWS GSH & Serum Urea	0.600	0.018
NWS AOPP & eGFR	−0.646	0.007
SWS UA & eGFR	−0.567	0.018
SWS UA & Proteinuria	0.576	0.015
SWS UA & ALP	0.632	0.010
SWS Albumin & eGFR	−0.552	0.027
SWS Albumin & Proteinuria	0.625	0.010
SWS MDA & PTH	−0.554	0.023
SWS AOPP & eGFR	−0.552	0.005
SWS AOPP & Proteinuria	0.490	0.015
SWS AOPP & Serum Urea	0.561	0.004

ALP, alkaline phosphatase; AOPP, advanced oxidation protein products; eGFR, estimated glomerular filtration rate using Schwartz formula; GSH, reduced glutathione; MDA, malondialdehyde; NWS, non-stimulated whole saliva; PTH, parathyroid hormone; SWS, stimulated whole saliva; UA, uric acid.

**Table 6:** Correlations between salivary and blood oxidative stress biomarkers in children with chronic kidney disease (CKD) and the controls.

Pairs of Variable	*r*	*P*
*Control group*		
NWS UA & plasma UA	0.436	0.016
NWS albumin & plasma albumin	0.682	0.010
NWS AOPP & plasma AOPP	0.591	0.010
SWS AOPP & plasma AOPP	0.495	0.029
*CKD children*		
NWS UA & plasma UA	0.668	0.005
NWS albumin & plasma albumin	0.566	0.018
SWS albumin & plasma albumin	0.483	0.015
NWS TAC & plasma TAC	0.633	0.004
SWS TOS & plasma TOS	0.531	0.023
NWS AOPP & plasma AOPP	0.665	0.015
SWS AOPP & plasma AOPP	0.550	0.004
NWS MDA & plasma MDA	0.518	0.016

**Table 7:** Receiver operating characteristic (ROC) analysis of salivary oxidative stress biomarkers of children with chronic kidney disease (CKD) and the controls.

Parameter	NWS					SWS				
	AUC	*P* Value	Cutt-Off	Sensitivity (%)	Specificity (%)	AUC	*P* Value	Cutt-Off	Sensitivity (%)	Specificity (%)
*Non-enzymatic antioxidants*										
UA (μg/mg protein)	0.82	0.0040	>4.03	64.71	66.67	0.93	<0.0001	>7.30	88.24	92.31
GSH (μg/mg protein)	0.75	0.0282	<1.42	66.67	66.67	0.84	0.0025	<1.26	80.00	83.33
Albumin (mg/mg protein)	0.79	0.0094	>1.55	81.25	83.33	0.94	0.0002	>1.55	93.33	90.91
*Enzymatic antioxidants*										
CAT (nmol H_2_O_2_/min/mg protein)	0.52	0.8823	>0.83	50.00	54.55	0.58	0.4472	<0.54	60.00	61.54
Px (mU/mg protein)	0.61	0.3097	<0.42	61.11	66.67	0.86	0.0007	>0.47	83.33	84.62
SOD (mU/mg protein)	0.79	0.0160	>0.31	71.43	72.73	0.74	0.0250	>0.14	72.22	69.23
*Total antioxidant/oxidant status*										
TAC (Trolox μmol/mg protein)	0.68	0.1074	<0.51	66.67	66.67	0.81	0.0050	>0.42	75.00	76.92
TOS (nmol H_2_O_2_Equiv/mg protein)	0.66	0.1385	>23.26	55.56	58.33	0.95	<0.0001	>25.90	88.89	84.62
OSI (TOS/TAC ratio)	0.59	0.46	>48.82	50.00	54.55	0.95	< 0.0001	>27.20	88.89	92.31
*Oxidative damage products*										
AGE (fluorescence/mg protein)	0.75	0.0223	>1.98	77.78	75.00	0.91	0.0001	>2.08	88.89	92.31
AOPP (nmol/mg protein)	0.92	0.0003	>37.85	81.25	81.82	0.98	<0.0001	>25.58	92.00	92.31
MDA (μmol/mg protein)	0.86	0.0006	>100.10	77.78	75.00	0.95	<0.0001	> 100.00	83.33	84,62

AGE, advanced glycation end products; AOPP, advanced oxidation protein products; AUC, area under curve; CAT, catalase; GSH, reduced glutathione; MDA, malondialdehyde; NWS, non-stimulated whole saliva; Px, salivary peroxidase; SOD, superoxide dismutase; SWS, stimulated whole saliva; TAC, total antioxidant capacity; TOS, total oxidant status; UA, uric acid.
